# Administration of BMSCs with Muscone in Rats with Gentamicin-Induced AKI Improves Their Therapeutic Efficacy

**DOI:** 10.1371/journal.pone.0097123

**Published:** 2014-05-13

**Authors:** Pengfei Liu, Yetong Feng, Chao Dong, Dandan Yang, Bo Li, Xin Chen, Zhongjun Zhang, Yi Wang, Yulai Zhou, Lei Zhao

**Affiliations:** 1 Department of Regeneration Medicine, School of Pharmaceutical Science, Jilin University, Changchun, P.R. China; 2 Key Laboratory of Regenerative Biology, Guangdong Provincial Key Laboratory of Stem Cell and Regenerative Medicine, Guangzhou Institutes of Biomedicine and Health, Chinese Academy of Sciences, Guangzhou, P.R. China; 3 Department of Laboratory Medicine, Second Clinical Medical College of Jinan University, Shenzhen People's Hospital, Shenzhen, P.R. China; 4 Department of Anesthesiology, Second Clinical Medical College of Jinan University, Shenzhen People's Hospital, Shenzhen, P.R. China; Rutgers - New Jersey Medical School, United States of America

## Abstract

The therapeutic action of bone marrow-derived mesenchymal stem cells (BMSCs) in acute kidney injury (AKI) has been reported by several groups. However, recent studies indicated that BMSCs homed to kidney tissues at very low levels after transplantation. The lack of specific homing of exogenously infused cells limited the effective implementation of BMSC-based therapies. In this study, we provided evidence that the administration of BMSCs combined with muscone in rats with gentamicin-induced AKI intravenously, was a feasible strategy to drive BMSCs to damaged tissues and improve the BMSC-based therapeutic effect. The effect of muscone on BMSC bioactivity was analyzed *in vitro* and *in vivo*. The results indicated that muscone could promote BMSC migration and proliferation. Some secretory capacity of BMSC still could be improved in some degree. The BMSC-based therapeutic action was ameliorated by promoting the recovery of biochemical variables in urine or blood, as well as the inhibition of cell apoptosis and inflammation. In addition, the up-regulation of CXCR4 and CXCR7 expression in BMSCs could be the possible mechanism of muscone amelioration. Thus, our study indicated that enhancement of BMSCs bioactivities with muscone could increase the BMSC therapeutic potential and further developed a new therapeutic strategy for the treatment of AKI.

## Introduction

Acute kidney injury (AKI) is the rapid deterioration of renal function. It can be induced by numerous insults, and has high morbidity and mortality rates [1]–[3]. The mortality rate of hospital-acquired AKI currently ranges from 30% to 80%, and recent dialysis techniques, such as pharmacological therapy and continuous renal replacement therapy, have no obvious effects on the overall mortality [4]–[6]. Thus, a novel therapeutic strategy should be developed to improve the survival outcomes of patients with AKI.

In recent years, great interest has shifted to stem cell-based therapy in the treatment of many diseases, such as diabetes [7], neural disease [8], and so on [9]–[12]. In the treatment of AKI, the therapeutic actions of stem cells in preventing and repairing damaged renal cells have been demonstrated by previous studies [13]–[15]. Different types of stem cells, such as amniotic fluid stem cells [16], hematopoietic progenitor cells [17], and kidney-derived mesenchymal stem cells[18], have been investigated, and their therapeutic effects in AKI treatment have been determined. Especially for the action of bone marrow derived mesenchymal stem cells (BMSCs), several studies have used BMSCs to treat AKI in animal models and their results showed that renal function and structure could be improved with the infusion of BMSCs [19]–[21]. BMSCs can be isolated from the bone marrow of patients. Compared with other stem cells, BMSCs are feasible in autologous treatment because of their source, number, and safety [21], [22].

The therapeutic mechanism of BMSCs comprises both differentiation-dependent mechanism [23], [24] and differentiation-independent mechanism [25]. However, which of these two mechanisms is more significant during the improvement in kidney function and structure remains unclear.

Besides further exploring the accurate mechanism, researchers are also interested in further refining the therapeutic action of BMSCs. Liu et al. found that BMSC transplantation combined with erythropoietin (EPO) injection was a novel and effective approach for AKI repairing [19]. They showed that the AKI microenvironment had a direct chemotactic effect on BMSCs, which could be further enhanced by EPO treatment. The activation of PI3K/AKT and MAPK in BMSCs and the increase in stromal cell-derived factor (SDF-1) levels in the AKI microenvironment were the possible mechanisms for the effect of EPO. However, the experimental events in their study were carried out *in vitro*, the function of EPO *in vivo* remained unknown. To further analyze the enhancement of EPO on the effects of stem cell-based therapy, Eliopoulos et al. reported that BMSCs, which were genetically enhanced to secrete EPO, could produce significant beneficial effects in AKI therapy [26]. They created mouse EPO-secreting BMSCs, which were implanted by intraperitoneal injection in allogeneic mice that were previously administered with cisplatin to induce AKI. Their results showed that EPO-BMSCs significantly improved the survival rate of implanted mice than normal BMSCs. Liu et al. investigated the effect of CXCR4 overexpression on BMSC migration to the kidney in AKI treatment [27]. CXCR4 gene-modified BMSCs (CXCR4-BMSCs) and normal BMSCs were prepared and transplanted into AKI mice. Their results showed that overexpression of the CXCR4 gene enhanced BMSC migration to the kidney after AKI. However, Gheisari et al. reported a different conclusion [28]. In their study, CXCR4 and CXCR7 were separately and simultaneously overexpressed in BMSCs with a lentiviral vector system, and the homing and renoprotective potentials of these cells were evaluated in the mouse model of cisplatin-induced AKI. They concluded that the overexpression of CXCR4 and CXCR7 receptors in BMSCs could not improve the homing and therapeutic potentials of these cells, and it could be due to severe chromosomal abnormalities in these cells during expansion *in vivo*. Therefore, gene-modified BMSCs are unstable during treatment, and they have yet to be used in clinical studies because of the associated laws and regulations. Thus, a novel strategy for the improvement of stem cell-based therapy should be developed.

In this study, muscone was used to optimize the therapeutic action of BMSCs by enhancing cell migration, proliferation, and secretory capacity. Natural muscone is obtained from musk, which is a ventral glandular secretion of the male musk deer, and is regarded as the main active ingredient of musk [29], [30]. Musk has been extensively used in Chinese medicine for thousands of years, and muscone is believed to hold less toxicity and side effects compared with musk, and also hold the function as a refreshing agent, promoting blood flow and detumescence [30], [31]. Therefore, muscone is widely used in clinical studies. Xie et al. first investigated the effect of muscone on BMSC migration *in vivo* [32], in which a rat model of skull defect was established through dental surgery. BMSCs combined with muscone were injected into skull-defect rats from the tail vein. Their results indicated that muscone could accelerate the migration of BMSCs to the injured area *in vivo*. However, whether muscone promotes cell migration in AKI model remains unclear. Our study showed that BMSCs combined with muscone was a feasible strategy in promoting BMSC migration, proliferation, and secretory capacity to repair kidney tissues in the rat model of gentamicin-induced AKI. This strategy could be used to develop a novel treatment mode for the preclinical study of AKI.

## Results

### Characterization of BMSCs and RTECs

**Figure 1:**
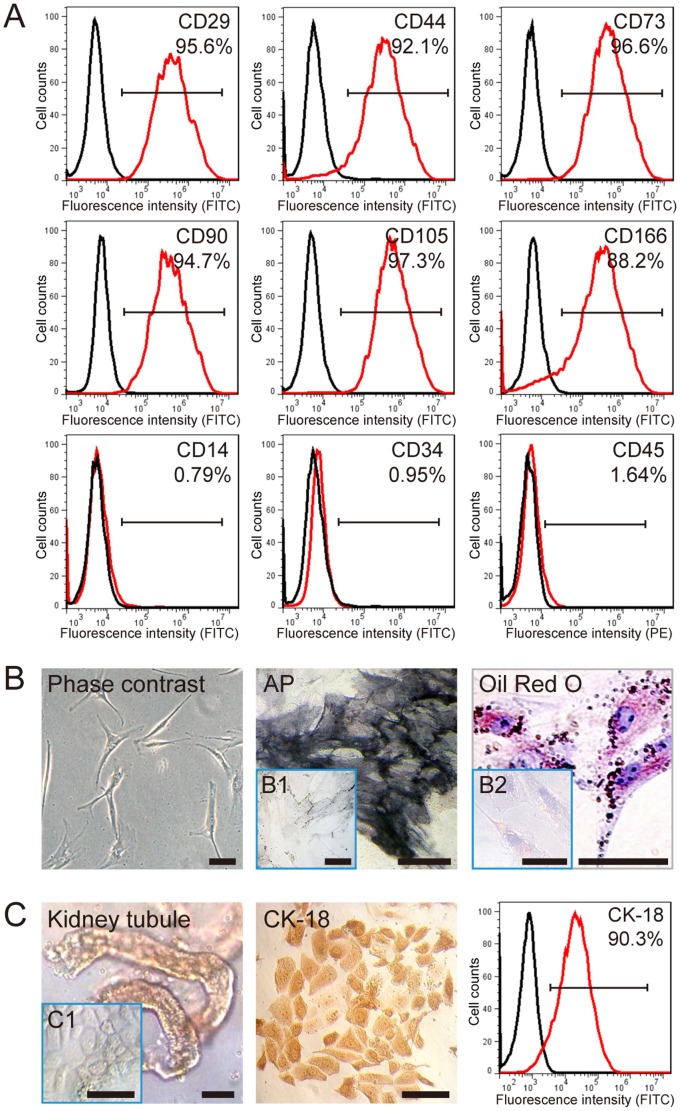
Characterization of BMSCs and RTECs. A: Immunophenotype of isolated BMSCs. Isolated rat BMSCs were characterized by FACS. BMSCs were positive for CD29, CD44, CD73, CD90, CD105, and CD166, and nearly negative for CD14, CD34, and CD45. B: Differentiation characteristics of BMSCs. The phase contrast of BMSCs is shown on the left. Osteogenic differentiation was detected by AP staining (middle), and adipogenic differentiation was visualized by Oil Red O staining of the lipid vesicles (right). BMSCs cultured with normal medium were used as the negative control group for each stain (B1 and B2). C: Immunophenotype of isolated RTECs. The appearance of kidney tubules is shown on the left and the confluent RTECs in culture were shown in C1. The isolated rat RTECs were characterized by immunohistochemistry (middle) and FACS (right), and they were positive for CK-18. All scale bars correspond to 200 µm.

The BMSCs isolated from Wistar rats were characterized by FACS for CD14, CD29, CD34, CD44, CD45, CD73, CD90, CD105, and CD166. The BMSCs used in our study were positive for CD29 (95.6%), CD44 (92.1%), CD73 (96.6%), CD90 (94.7%), CD105 (97.3%), and CD166 (88.2%), and nearly negative for CD14 (0.79%), CD34 (0.95%) and CD45 (1.64%), which were regared as the specific markers of hematopoietic cells (Figure 1A). The BMSCs displayed a spindle-shaped “fibroblast” appearance. They were successfully differentiated into adipocytes and osteoblasts, as demonstrated by Oil Red O staining and positive staining with alkaline phosphatase (AP), respectively (Figure 1B). In the negative control group, the BMSCs cultured with normal medium were used for each stain, and could not be stained with Oil Red O or AP kit (Figures 1B1 and 1B2). In the process of RTECs isolation, kidney tubules were first separated from kidney tissues (Figure 1C). With further culture *in vitro*, the kidney tubules gradually attached to the plastic surface. The RTECs grew out from the tubules and continued to pave stone, resembling growth (Figure 1C1). After one passage, the RTECs were characterized by FACS and immunohistochemistry for CK-18, and a positive stain was observed (Figure 1C). These results confirm the purity and differentiation ability of the cells used in our culture system.

### Effect of muscone on BMSC and RTEC proliferation and secretion

**Figure 2:**
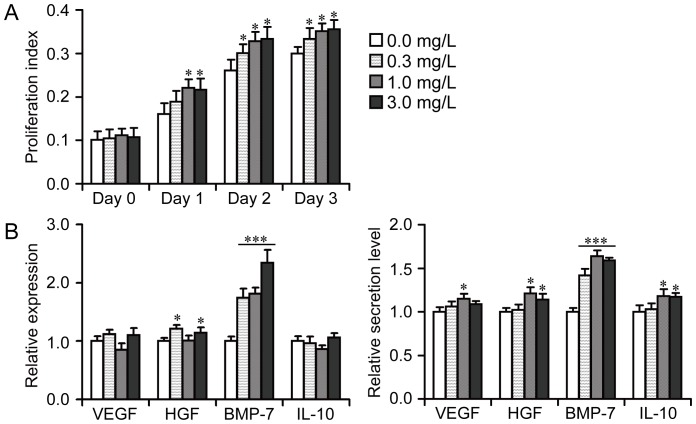
Effect of muscone on BMSC bioactivity *in vitro*. A: Effect of muscone on BMSC proliferation. Proliferation index (the absorbance of experimental group − the absorbance of blank group) was measured using CCK-8. B: Effect of muscone on BMSC secretion. To evaluate the secretory function, the secretion or cytokine expression level of normal BMSCs without any muscone treatment was 1.0 for each cytokine. Expression of the cytokines in BMSCs detected with qPCR is shown on the left, and the BMSC secretory function evaluated using ELISA is shown on the right. Similar results were obtained in at least three independent experiments. Results were expressed as mean ± SEM. A *t*-test was used to compare the various groups, and *P*<0.05 was considered statistically significant. *P<0.05 compared with normal BMSC group without any muscone treatment; ***P<0.001 compared with normal BMSC group without any muscone treatment.

The treatment of muscone at different concentrations (0.3, 1.0, and 3.0 mg/L) enhanced the proliferative ability of BMSCs to some degree (Figure 2A). The absolute values in proliferation indexes of each group were shown in Table S1, and the fold change can be counted based on those values, showing up to 1.37 fold increases with the treatment of muscone compared with the normal cells. The function of BMSCs in secreting renal protective cell factors (VEGF, HGF, BMP-7, and IL-10) was further analyzed. Real-time qPCR showed that BMP-7 expression in BMSCs was upregulated by muscone than that in normal BMSCs (Figure 2B). These results were further confirmed using ELISA. However, other cell factors secreted by BMSCs in ELISA still demonstrated a modest increasing tendency (Figure 2B). The effect on RTEC proliferation and secretion was also evaluated in our study. No obvious variation was observed in RTEC proliferative activity during 3 days of culture *in vitro* (Figure S1A). qPCR and ELISA showed that the expression of renal protective cell factors (HGF, TGF-β, TIMP-1, and ET-1) in RTECs and the function of RTECs to secrete these cell factors in each treatment group were similar to those of the normal group (Figure S1B).

### Evaluation of rat AKI model

In our study, the AKI model was induced by gentamicin in adult male Wistar rats. To confirm the rat AKI model, urine and serum samples were collected on day 8 before treatment. The levels of N-acetyl-beta-glucosaminidase (NAG) and lysozyme (LZM) in urine normalized by urinary creatinine (Table S2), as well as creatinine and urea nitrogen in serum, were measured in the normal and AKI model groups. These indices in the model group presented significantly higher levels than those in the normal group (NAG: 63.1±6.3 U/L vs. 13.8±2.7 U/L; LZM: 302.4±55.5 U/ml vs. 32.7±2.7 U/ml; creatinine: 65.2±9.9 µM vs. 21.2±7.7 µM; and urea nitrogen: 26.46±4.1 mM vs. 9.5±2.1 mM, *P*<0.05). These findings demonstrated that the rat model displayed characteristics of AKI disease (Table 1).

**Table 1:** Change of biochemical variables in serum or urine during the therapy process.

	Characteristics of AKI model in urine or serum				Biochemical variables in urine after therapy			Biochemical variables in serum after therapy		
	NAG(U/L)	LZM(U/ml)	Creatine(µM)	UN(mM)	NAG(U/L)	LZM(U/ml)	UN(mM)	NAG(U/L)	Creatine(µM)	UN(mM)
normal group	13.8±2.7	32.7±2.7	21.2±7.7	9.5±2.1	17.4±3.2	34.2±4.2	33.1±3.1	17.9±3.1	19.9±6.7	7.7±2.1
model group	63.1±6.3^*^	302.4±55.5^*^	65.2±9.9^*^	26.5±4.1^*^	62.7±17.2	406.7±71.4	15.7±5.6	58.3±5.6	78.4±6.5	28.1±4.6
positive drug group					19.3±5.6^*^	249.1±32.6^*^	24.3±4.9^*^	16.3±3.2^*^	22.1±4.3^*^	26.3±4.4
muscone group					23.0±5.8^*^	278.1±66.6^*^	16.1±4.2	17.8±5.7^*^	42.2±3.2^*^	27.3±6.2
BMSCs group					20.1±7.1^*^	273.5±82.3^*^	25.1±2.6^*^	19.7±4.3^*^	33.2±3.8^*^	26.4±5.3
combined group					17.8±5.3^*^	254.2±59.4^*^	30.8±3.5^*#^	19.5±5.3^*^	26.6±4.3^*#^	28.7±4.8

To assay the characteristics of rat AKI model, NAZ and LAM in urine, as well as creatinine and urea nitrogen (UN) in serum, were detected on day 7. *P<0.05 compared with the normal group. To analyze the therapeutic effect, urine and serum samples of each group were collected from each group on day 15 after animals were sacrificed. The levels of UN, NAZ, and LAM in urine, as well as creatinine, UN, and NAG in serum, were measured. *P<0.05 compared with the model group; #*P*<0.05 compared with the muscone and BMSC groups.

### Effect of treatment on kidney weight coefficient

**Figure 3:**
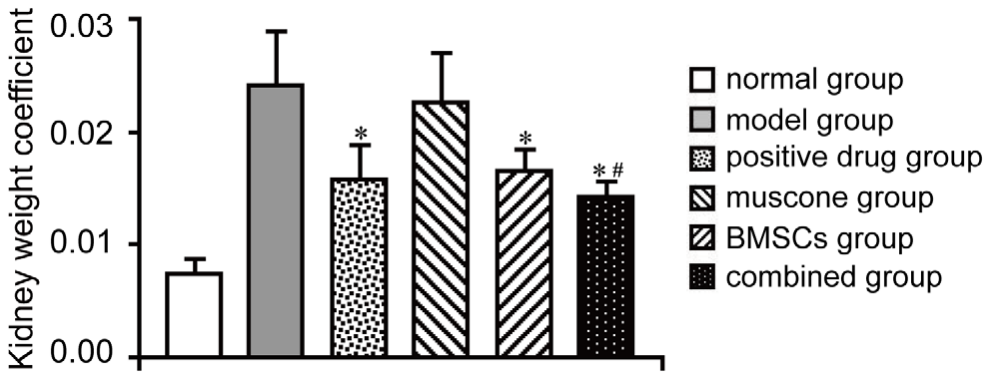
Level of kidney weight coefficients in each group. *P<0.05 compared with the model group; #*P*<0.05 compared with the muscone and BMSC groups.

To evaluate the therapeutic action on the accrementition and enlargement of the entire kidney, the kidney weight coefficient was determined in each group. We found that the kidney weight coefficient was below 0.01 in the normal group, whereas that of the model group exceeded 0.02. The coefficients of the other groups decreased in varying degrees with therapy (Figure 3). Both the positive drug and BMSCs had an effect to release the enlargement of kidney, and the kidney weight coefficient of the BMSC group was similar to that of the positive drug group. In the combined group, the BMSCs and muscone were given simultaneously, and the therapeutic effect of BMSCs was further enhanced with muscone treatment, and an optimal protective function in inducing the accrementition of kidney tissues was observed in the combined group. However, no significant therapeutic effect was observed in the muscone group, which indicated that muscone did not have an obvious therapeutic function in AKI.

### Effect of treatment on biochemical variables in urine and serum

The levels of urea nitrogen, NAG, and LZM in urine normalized by urinary creatinine (Table S2), as well as creatinine, urea nitrogen, and NAG in serum, were measured using a Biochemistry Autoanalyzer. LZM showed a statistically significant difference between the model and treatment groups. The levels of NAG and urea nitrogen in the treatment groups were similar to the normal levels, especially urea nitrogen in the combined group (Table 1). The injection of BMSCs and muscone significantly decreased NAG and creatinine in serum (*P*<0.05). The creatinine level in the combined group was the most similar to that of the normal group. However, no amelioration was found in urea nitrogen in serum (Table 1).

### Effect of treatment on renal histology

**Figure 4:**
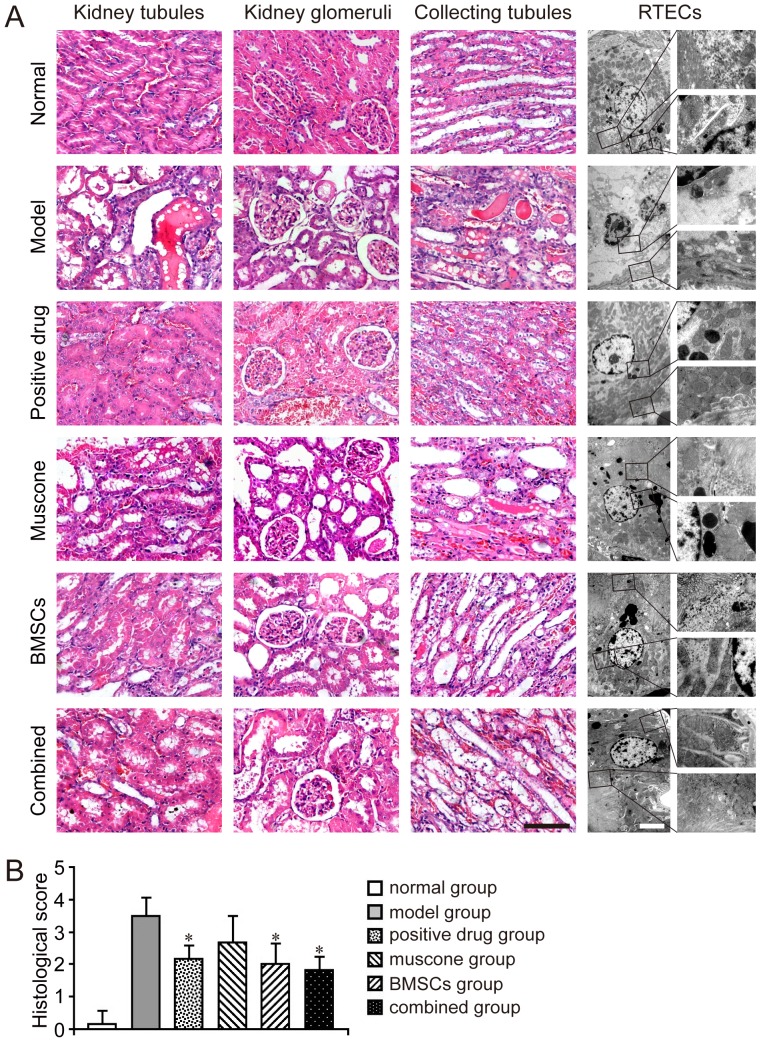
Detection of pathological changes in each group. A: Pathological changes of each group. Pathological changes in the kidney tubules, kidney glomeruli, and collecting tubules were observed under a light microscope using H&E staining. Dilatation and necrosis in the kidney tubules and collecting tubules of the model group were observed in various degrees. In the combined and BMSC groups, nearly no effusion was observed in the kidney tubules or collecting tubules, and both tubules remained slightly expanded compared with the normal group. Ultrastructures of RTECs were further observed under TEM. The RTECs in the normal group had minimal heterochromatin and numerous organelles in the cytoplasm. In the AKI model, the RTECs showed an apoptotic appearance with deformed nuclei, more heterochromatin, and fewer organelles. Positive drug or muscone treatment resulted in numerous mitochondria and few cytolysosomes in the RTEC cytoplasm. The RTECs of both the BMSC and combined groups were restored well, and showed no significant difference. Scale bar of the phase observed through H&E staining corresponds to 100 µm, and scale bar of the phase observed under TEM corresponds to 4 µm. B: Histological score of each group. Results are expressed as mean ± SEM. A *t*-test was used to compare the various groups. *P<0.05 compared with the model group.

To evaluate the therapeutic effect of BMSCs and muscone in the AKI model, the pathological changes in the kidney tubules, kidney glomeruli, and collecting tubules in each group were observed by H&E staining. The typical pathological changes in AKI induced by gentamicin were mainly reflected in the kidney tubules and collecting tubules. The kidney glomeruli in all groups were similar to one another. Notable damage, including tubular necrosis, dilatation, and effusion in the kidney tubules and collecting tubules, was observed in the model group compared with the normal group. Amelioration of various degrees was observed in the treatment group, and both the BMSC and combined groups showed better restoration than the muscone group, and there is no difference between the combined group (the BMSCs and muscone were given simultaneously) and the BMSCs group in terms of histopathology. The BMSC and combined groups exhibited fewer necrotic and dilated tubules and less effusion in the tubules, especially in the recovery of collecting tubules. The muscone group still showed therapeutic effects than the model group (Figure 4A). A histological scoring system was used to further evaluate kidney tissue morphology. The histological score of normal group was below 1, whereas that of the model group exceeded 3. The score of other groups could be decreased with the treatment, and there was no significant difference between the BMSC and the combined groups (Figure 4B).

Samples of each group were observed under TEM to analyze the ultrastructures of RTECs. The RTECs in the normal group had clear ultrastructures with minimal heterochromatin and numerous organelles in the cytoplasm. In the model group, the RTECs exhibited noticeable apoptosis with fewer organelles, deformed nuclei, and markedly more heterochromatin. The ultrastructures of RTECs in the treatment groups were improved in some degree. Abundant mitochondria and some cytolysosomes were observed in the cytoplasm of the positive drug group. After treatment with BMSCs or muscone, the RTEC structures exhibited better restoration with abundant organelles and some cytoplasmic vacuoles. However, no significant difference was found between the BMSC and combined groups (Figure 4A).

### Cell apoptosis assay

**Figure 5:**
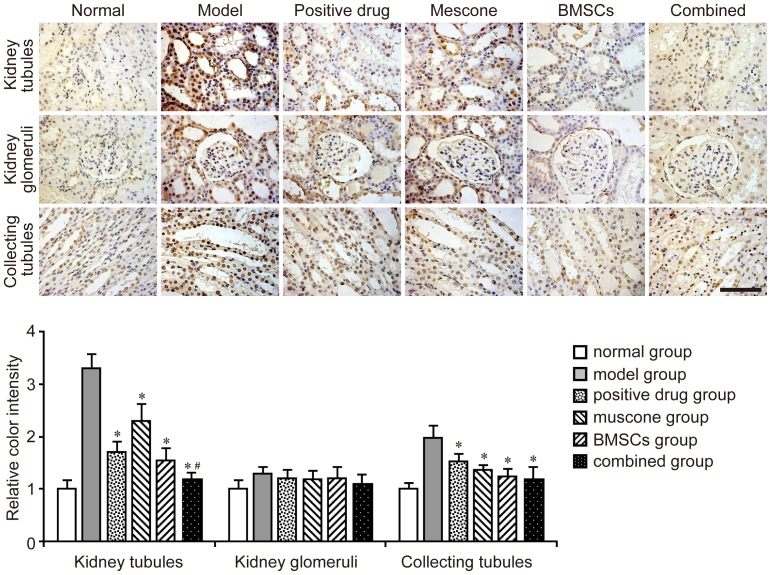
Evaluation of cell apoptosis in each group using TUNEL staining. Images of TUNEL staining in the kidney tubules, kidney glomeruli, and collecting tubules are shown for each group. The color intensity was measured using Motic Image Advanced 3.2. The color intensity of the normal group was 1.0, and the relative color intensity of the other groups was evaluated. Similar results were obtained in at least three independent experiments. Results are expressed as mean ± SEM. A *t*-test was used to compare the various groups, and *P*<0.05 was considered statistically significant. *P<0.05 compared with the model group; #*P*<0.05 compared with the muscone and BMSC groups. Scale bar corresponds to 100 µm.

TUNEL assay was used to detect apoptosis in renal cells in the kidney sections. The number of TUNEL-positive cells in both the kidney tubules and collecting tubules markedly increased in the model group but decreased in the treatment group (Figure 5). Motic Image Advanced 3.2 software showed that the color intensity of the combined group (the BMSCs and muscone were given simultaneously) was lighter than that of the BMSC or muscone group in the kidney tubules. However, the combined group did not show a superior improvement in the therapeutic effect in the collecting tubules. TUNEL assay detected no significant difference in cell apoptosis of the kidney glomeruli between the model and other groups (Figure 5).

**Figure 6:**
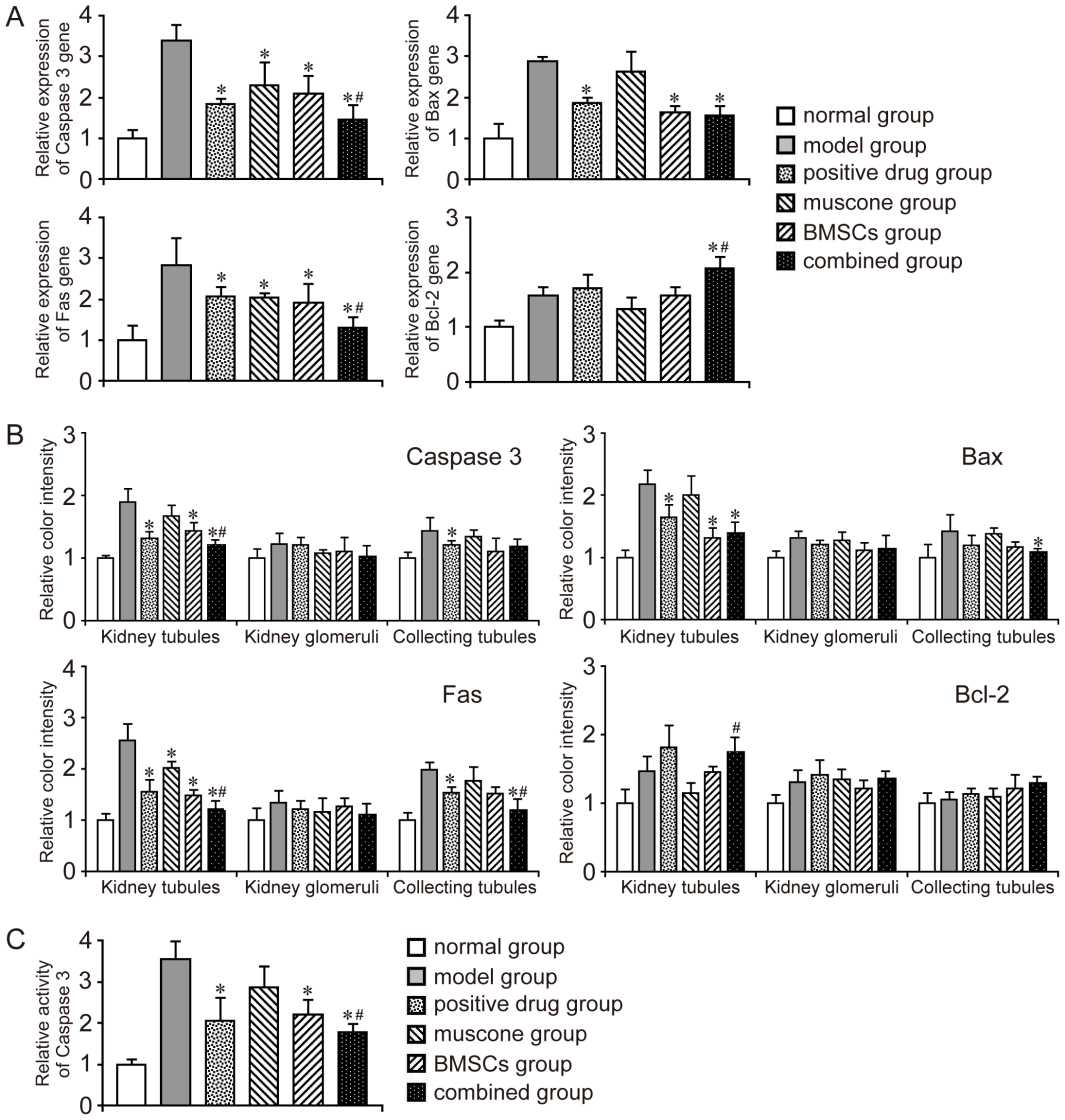
Expression of apoptotic genes and anti-apoptotic gene in the kidney tissues of each group. A: Detection of apoptotic genes (Caspase 3, Fas, and Bax) and anti-apoptotic gene (Bcl-2) expression using qPCR. The gene expression level in the normal group was 1.0, and the relative gene expression level of each group was further evaluated. B: Detection of apoptotic genes and anti-apoptotic gene expression using immunohistochemistry. The color intensity was further measured using Motic Image Advanced 3.2. The color intensity of the normal group was 1.0, and the relative color intensity of the other groups was further evaluated. C: Detection of activated Caspase3 in kidney tissues. Similar results were obtained in at least three independent experiments. Results are expressed as mean ± SEM. A *t*-test was used to compare the various groups, and *P*<0.05 was considered statistically significant. *P<0.05 compared with the model group; #*P*<0.05 compared with the muscone and BMSC groups.

The expression levels of apoptotic genes (Caspase 3, Fas, and Bax) and anti-apoptotic gene (Bcl-2) in the kidney tissue of each group were detected. Real-time qPCR showed that the expression levels of apoptotic genes in the model group were much higher than those in the normal group. Most treatments were efficient in inducing anti-apoptosis. By contrast, muscone did not significantly downregulate Bax expression, and the combined group showed reduced expression levels of Caspase 3 and Fas (Figure 6A). The expression of anti-apoptotic gene (Bcl-2) was much higher in the combined group than that in the single therapy and model groups (Figure 6A).

The expression levels of apoptotic genes (Caspase 3, Fas, and Bax) and anti-apoptotic gene (Bcl-2) in kidney tissues were also detected using immunohistochemistry. A representative set close to the average level of each group is shown in Figure S2, and most of the results were similar to those of qPCR. Gene expression levels in the kidney tubules, kidney glomeruli, and collecting tubules were analyzed. Motic Image Advanced 3.2 software was used to measure the color intensity of each group. No noticeable difference in gene expression was observed between the model and other groups in the kidney glomeruli. Both the positive drug and combined groups showed a modest therapeutic effect on cell apoptosis in the collecting tubules. The differences among each group were mainly reflected in the kidney tubules. All apoptotic genes were downregulated in the treatment groups, except the muscone group. The combined group exhibited lower expression of apoptotic genes (Caspase 3 and Fas) and higher expression of anti-apoptotic gene (Bcl-2) than those in the BMSC or muscone group (Figure 6B). In addition, the activated Caspase3 in the kidney tissue was also determined by the kit. The result was similar to that of qPCR and the combined group showed better therapeutic action on inhibiting cell apoptosis, compared with the single therapy (Figure 6C).

### Inflammatory components assay

**Figure 7:**
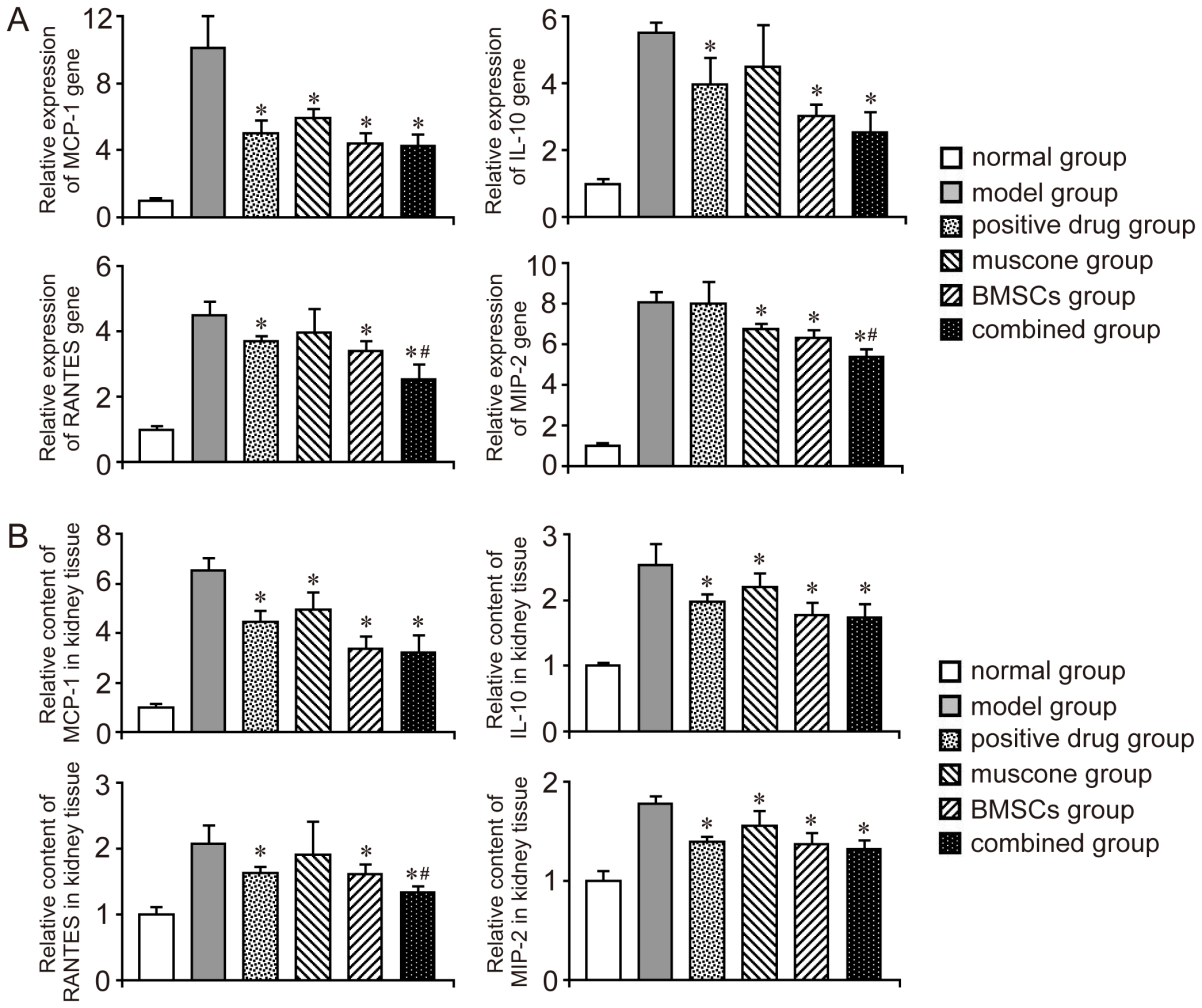
Evaluation of inflammatory reaction in the kidney tissues of each group. A: Detection of inflammatory component using qPCR. B: Detection of inflammatory component using ELISA. Similar results were obtained in at least three independent experiments. Results are expressed as mean ± SEM. A *t*-test was used to compare the various groups, and *P*<0.05 was considered statistically significant. *P<0.05 compared with the model group; #*P*<0.05 compared with the muscone and BMSC groups.

Expression of MCP-1, IL-10, RANTES and MIP-2 in RNA level and protein level were examined in our study. All of the inflammatory components were down-regulated with the therapy in different degree (Figure 7A, 7B). Both the positive drug and muscone showed therapeutic effect on the regulation of some inflammatory components. Compared with other group, qPCR results showed the combined group (the BMSCs and muscone were given simultaneously) hold better therapeutic action on down-regulating RANTES and MIP-2 (Figure 7A), and the effect on RANTES could be further confirmed in the ELISA assay (Figure 7B).

### Effect of muscone on BMSC migration

**Figure 8:**
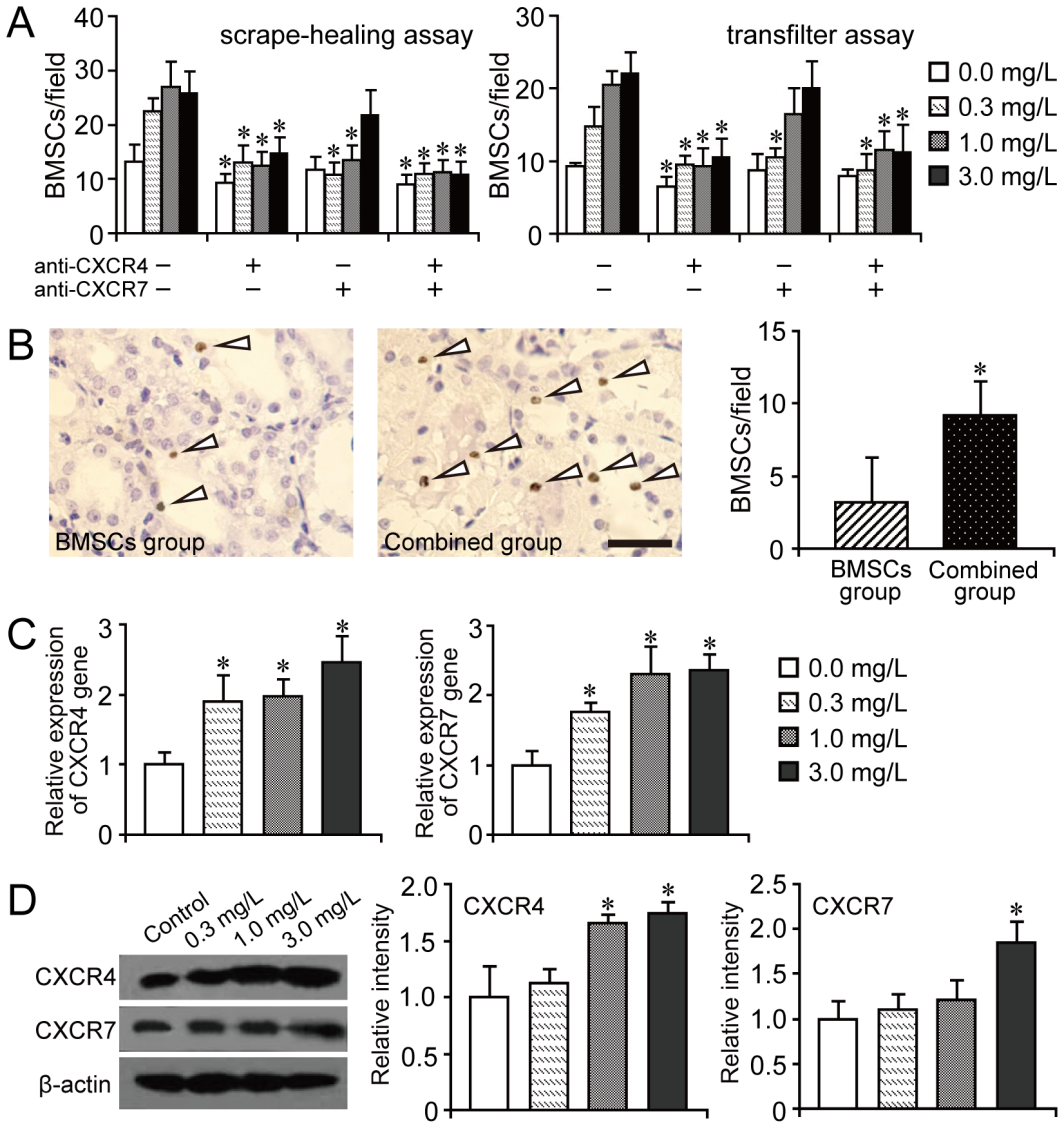
Effect of muscone on BMSC migration capacity. A: Quantification of the BMSC migration capacity using scrape-healing assay and transfilter assay. The number of migrating BMSCs was counted in each field. BMSCs exposed to CXCR4 and CXCR7 neutralizing antibodies were used for the both assay, and the promigratory effect of muscone on BMSC was abrogated in different degree. *P<0.05 compared with the corresponding group without the treatment of CXCR4 or CXCR7 neutralizing antibody. B: Enhancement of BMSC migration *in vitro*. Representative images of BrdU-positive cells in the BMSC and combined groups. The number of BrdU-positive cells was further counted in each field. Scale bar corresponds to 30 µm. C and D. Expression of CXCR4 and CXCR7 in BMSCs with or without muscone treatment detected using qPCR and western blot, respectively. Similar results were obtained in at least three independent experiments. Results are expressed as mean ± SEM. A *t*-test was used to compare the various groups, and *P*<0.05 was considered statistically significant. *P<0.05 compared with the normal BMSC group without any muscone treatment.

To investigate the effect of muscone on BMSCs and explain the improved therapeutic effect in the combined group, the migratory ability of BMSCs was detected *in vitro* and *in vivo*. BMSC healing capacity was quantified using a scrape-healing assay in which cells were scratched. Muscone treatment could enhance the migration of BMSCs toward the injured area and reduce the scratch surface area. The number of BMSCs migrating to the injured area was counted in the observed field, and more cells were observed in the injured area with muscone treatment (Figure 8A). An *in vitro* injury-migration model was then applied as a reference [33] based on a transwell system consisting of BMSCs co-cultured with cisplatin-injured RTECs. BMSC migration from the upper chamber across the membrane to the cisplatin-damaged RTECs could be enhanced with muscone treatment compared with that without any treatment (Figure 8A). Preconditioned cells co-cultured with untreated RTECs or without RTECs did not exhibit significant migratory activity (Figure S3A). To evaluate migration *in vivo*, BrdU-labeled BMSCs were detected using immunohistochemistry, and the combined group had more labeled BMSCs than the BMSC group (Figure 8B).

To investigate the molecular mechanism by which cell migration is promoted, we detected the expression of CXCR4 and CXCR7 in the treated BMSCs using qPCR and western blot, respectively. CXCR4 and CXCR7 had pivotal functions in cell migration and proliferation. The qPCR results showed that muscone increased the expression of both CXCR4 and CXCR7 (Figure 8C). These results were similar to those of western blot. However, low muscone concentration could not increase the expression of those genes at a protein level (Figure 8D). To further define the biological significance of muscone-mediated CXCR4 and CXCR7 upregulation, we performed scrape-healing assay and transfilter assay in the presence of a neutralizing antibody. Our results showed that the functional blocking of CXCR4 or CXCR 7 could abrogate the promigratory effect of muscone on BMSC (Figure 8A).

The transfilter assay was also tried to be performed after a shorter incubation time (8 h, 12 h and 24 h) to avoid the obvious cell proliferation. However, no significant difference existed among each group under the circumstances (8 h and 12 h), and the obvious difference could be observed after 24 h (Figure S3B). This result indicated that enough time for the incubation could be necessary in the transfilter assay. Thus, to further investigate the influence of cell proliferation on the migration, BMSCs treated with mitomycin C were used for transfilter assay in our study. However, the results showed that there was no significant difference between the normal BMSCs and the mitomycin C treated BMSCs in the migration assay (Figure S3C). This indicated that the modest function to promote cell proliferation of muscone or cell proliferation by themselves, would not affect the migration in our transfilter assay system which only contained 2% FBS, though more cells would affect the migratory assay results.

## Discussion

BMSCs have been reported to preserve renal function in various AKI models [27], [34], [35]. Both the differentiation-dependent and differentiation-independent mechanisms have functions in the therapy process, but increasing reports have indicated that the differentiation-independent mechanism has a more important function during treatment *in vivo* [23], [24], [35]. Chen et al. [35] evaluated the organ bio-distributions of transplanted BMSCs, and correlated the survival of transplanted cells. They found that BMSCs were largely localized in pulmonary capillaries after intravenous administration. Moreover, they reported that only a minute fraction of BMSCs could enter the kidneys, exhibiting transient survival. The development of novel strategies in enhancing cell homing to target tissues is regarded as a prerequisite for the success of BMSC-based systemic therapies. Xinaris et al. showed that the preconditioning of BMSCs with IGF-1 before infusion could improve cell migration and restore normal renal function after AKI. They demonstrated that promoting BMSC migration could increase their therapeutic potential, and indicated a novel therapeutic paradigm for organ repair [33].

The present study reported a novel approach to optimize BMSC engraftment efficiency and increased its therapeutic effects based on the combination with muscone in systemic infusion. Muscone improved BMSC engraftment in the injured kidney, as well as other bioactivities, such as cell proliferation and secretion. This function possibly improved the therapeutic effect of the combined group in some respects, including kidney weight coefficient, some biochemical variables, cell apoptosis and inflammation. Muscone also showed a therapeutic effect in the AKI model through anti-apoptosis, anti-inflammation and amelioration in pathological changes. However, the kidney weight coefficient did not significantly decrease with muscone treatment. This result may be attributed to the amount of effusion in kidney tissues, which was observed by H&E staining.

Furthermore, dexamethasone were used as the positive drug in our study and also showed a therapeutic function to treat the nephrotoxic injury. As a kind of glucocorticoids, dexamethasone was widely used in the clinical studies for the treatment of kidney injury [36]–[39]. In succession, its therapeutic function for different kidney injury animal models (cyclosporine A nephrotoxicity injury, ischemia/reperfusion injury, uranyl nitrate induced injury, endotoxin induced injury and gentamicin nephrotoxic injury) has been demonstrated, and associated mechanisms, such as ameliorating microvascular oxygenation and stabilizing TRPC6 expression and distribution, were also investigated by several groups [34], [40]–[45]. Therefore, it was chosen as the positive drug in our study. Even though dexamethasone showed some therapeutic action against AKI, it was still not as effective as BMSCs or the combined group in some respects.

The pathological changes in the kidney tubules, kidney glomeruli, and collecting tubules in each group were observed. Typical changes in AKI induced by gentamicin were mainly reflected in the kidney tubules and collecting tubules. However, the degree of cell apoptosis in the kidney tubules was more serious than that in the collecting tubules. Thus, the kidney tubules were the main damaged area in the AKI model induced by gentamicin. The combined group showed superior effects, but no significant difference in pathological changes was observed compared with the BMSC group. This result was similar to our previous study [34]. This phenomenon might be due to the unsuitable dosage of the muscone and BMSCs, and only one dosage was tested in our study after all. Therefore, further investigations on the dosage or other factors are needed to improve the therapeutic effect on pathological changes. Our previous study investigated the therapeutic effect of BMSCs combined with vitamin E against AKI in rats, and the combined treatment was better than BMSCs or vitamin E alone. However, the two treatments may function independently from each other. Muscone, which has been used in clinical studies for years, was used in this study to improve the therapeutic effect of BMSCs by enhancing cell migration and proliferation. This feasible therapeutic approach may produce better effects in future clinical studies.

Herein, several inflammatory components (MCP-1, IL-10, RANTES and MIP-2) in the kidney tissues of each group were analyzed with qPCR and ELISA. In these inflammatory components, IL-10 is capable of inhibiting synthesis of pro-inflammatory cytokines. For the compensation *in vivo*, IL-10 was up-regulated together with other pro-inflammatory cytokines, and down-regulated when The inflammatory reaction was relieved gradually. Our study had showed that muscone had no ability to increase the expression or secretion of IL-10 in BMSCs (Figure 2). The detection of IL-10 *in vivo* further confirmed no obvious difference existed between the BMSCs group and combined group (Figure 7). Even though our results showed that the combined group hold better therapeutic action on down-regulating RANTES and MIP-2 than the BMSCs group, the mechanism still need further exploration.

To further analyze the mechanism by which muscone promotes BMSC migration and proliferation, we detected the expression of CXCR4 and CXCR7 in BMSCs treated with muscone. Several groups have reported that the SDF-1/CXCR4 axis is a pivotal mediator of migration, proliferation, and survival of BMSCs [33], [46]–[49]. For most cell therapy experiments, expanding the cells *in vitro* is unavoidable. However, some reports showed that CXCR4 expression declined after several passages in the culture process [46], [50], which possibly decreased the homing and engraftment potentials of stem cells. Thus, the upregulation of CXCR4 expression on the stem cell surface should be an effective strategy to overcome this limitation. Although SDF-1 was originally assumed to signal exclusively through the chemokine receptor, CXCR4, another research identified CXCR7 as a second SDF-1 receptor [51], which interacted with CXCR4 and modulated its functions. Mazzinghi et al. investigated the function of CXCR4 and CXCR7 in renal progenitor cells [52]. They reported that both receptors were crucial for the homing and therapeutic potentials of these cells, and showed that CXCR7 was more involved in cell survival and adhesion to endothelium, whereas CXCR4 was involved in cell chemotaxis. In the present study, both CXCR4 and CXCR7 expression were upregulated in the BMSCs with muscone treatment, which could explain the function of muscone in promoting cell migration and proliferation.

In this study, the BMSCs and muscone were given simultaneously in rats with gentamicin-induced AKI. BMSCs preconditioned with muscone (3.0 mg/L, for 36 h) were also used to treat AKI rats in our previous research, but the therapeutic effect was not enhanced compared with the normal BMSC group, such as the improvement of kidney weight coefficient, biochemical variables in urine and serum, and cell apoptosis (data not shown). This result indicates the necessity of long-term treatment with muscone to enhance the therapeutic effect of BMSCs against AKI *in vivo*.

### Conclusions

In our study, muscone enhanced the therapeutic action of BMSCs by promoting cell proliferation, secretion, and migration. This finding could be used as a novel therapeutic approach for AKI or other diseases in the field of regenerative medicine through anti-apoptosis, anti-inflammation and amelioration in some biochemical variables. The mechanism was preliminarily investigated, and the expression of CXCR4 and CXCR7 was up-regulated in BMSCs after muscone treatment. However, the combined group showed no increase in pathological changes or some physiological indices. Thus, the optimal medication used in stem cell-based therapy for AKI should be further explored in different animal models of AKI, even in clinical studies.

## Methods and Materials

This study was carried out in strict accordance with the recommendations in the Guide for the Care and Use of Laboratory Animals of the National Institutes of Health. The protocol was approved by the Committee on the Ethics of Animal Experiments of Jilin University. All operations were performed under sodium pentobarbital anesthesia, and all efforts were made to minimize suffering.

### Isolation and characterization of BMSCs and renal tubular epithelial cells (RTECs)

Whole bone marrow was collected from the tibia and femur of a male Wistar rat weighing 120 g to 150 g. BMSCs were isolated from the whole bone marrow through density gradient centrifugation. The isolated cells were cultured in DMEM/F12 medium (Gibco, USA) containing 10% fetal bovine serum (FBS; Gibco), penicillin/streptomycin (100 U/mL to 0.1 mg/mL; Hyclone, USA), and 2 mM L-glutamine (Gibco, USA). The cells were incubated in a humidified incubator with 5% CO_2_ at 37°C. The media were changed every other day. The adherent cells were passaged by a dilution of 1∶3 once every 4 days or 5 days until passage 4, when a portion of the resulting BMSCs was prepared for phenotypic analysis by flow cytometry. The cells were then screened for CD14, CD29, CD34, CD44, CD45, CD73, CD90, CD105, and CD166. BMSCs were differentiated into adipocytes and osteocytes as previously described [53]. The rat RTECs were collected and cultured as described [34], [54], and screened for CK-18 after one passage with flow cytometry and immunohistochemistry. This operation was similar to that in our previous research [34].

### Effect of muscone on BMSC and RTEC proliferation and secretion

BMSCs and RTECs were planted in 96-well plates at 1×10^3^ and 3×10^3^ cells per well, respectively. The muscone (Santa, USA) concentrations used in the culture system were 0.3, 1.0, and 3.0 mg/L. Cell proliferation was detected at day 0, day 1, day 2, and day 3. The DMEM/F12 medium containing 10% FBS was used as the blank control group, and the proliferation index of each group was determined using the CCK-8 method (Dojindo, Japan) according to the manufacturer's instructions [34], which allowed sensitive colorimetric assays for the determination of the number of viable cells in cell proliferation. In brief, 10 µL of CCK-8 solution was added into each well (containing 100 µL of medium), and cultured for 1 h to 2 h at 37°C. The absorbance of each group at 450 nm was detected (n = 4) and it was directly proportional to the number of living cells. The proliferation index  =  the absorbance of experimental group − the absorbance of blank group, was used to measure cell proliferation in our study. After 2 days of culture, the medium of each group was collected for secretory cell function analysis. Vascular endothelial growth factor (VEGF), hepatocyte growth factor (HGF), bone morphogenetic protein-7 (BMP-7), and interleukin-10 (IL-10), which were secreted by BMSCs and relieved kidney injury, were measured using an ELISA Kit (Abcam, United Kingdom) following the manufacturer's instructions. The cells were collected for real-time quantitative PCR (qPCR) analysis. A similar operation was used to evaluate the effect of muscone on RTEC secretion [HGF, transforming growth factor-β (TGF-β), tissue inhibitor of metalloproteinase-1 (TIMP-1), and endothelin-1 (ET-1)].

### Flow cytometry

BMSCs or RTECs were dissociated into single cells with 0.25% trypsin, further fixed and permeated with Fixation Buffer (BD) and Perm/Wash Buffer (BD), respectively, and prepared at a concentration of 1.0×10^5^ cells in 100 µL of PBS. The antibodies, including CD14 conjugated to FITC (Biorbyt, UK), CD29 conjugated to FITC (BD, USA), CD44 conjugated to FITC (BD), CD45 conjugated to PE (BD), and CD90 conjugated to FITC (BioLegend, USA), were added and incubated for 30 min at 4°C. After two washes in PBS, cells were acquired and analyzed by FACScalibur (BD Bioscience). The antibodies, including CK-18 (Bioss, China), CD73 (BD), CD105 (Boster, China), CD34 (Abcam, United Kingdom), and CD166 (Santa), were added. After 30 min of incubation at 37°C, the cells were washed three times with PBS and incubated with FITC-conjugated goat anti-rabbit or donkey anti-goat IgG (Invitrogen, USA) for 30 min at 37°C. After two washes in PBS, cells were obtained and analyzed.

### Preparation and treatment of AKI model

Male Wistar rats, weighing 250 g to 300 g, were used in the experiment. All rats were housed in a room with constant temperature room with a 12 h dark-12 h light cycle and fed a standard diet. The method used to prepare or treat the AKI model is shown in Figure S4. In brief, animals were divided into six groups (six rats per group), namely, normal group, AKI model group, positive drug group, muscone group, BMSC group, and BMSCs combined with muscone group (combined group). To generate a rat model of AKI for testing the BMSCs and muscone *in vivo*, the model was induced by gentamicin (140 mg/kg/day for 7 days, i.p.) in rats. At day 8, BMSCs were administered by intravenous injection of 3.3×10^6^ cells/kg combined with muscone (75 mg/kg), whereas dexamethasone (0.08 mg/kg) was used as a positive control drug for 7 d. To trace the cells *in vivo*, BMSCs were labeled with 10 µM BrdU (Sigma). The normal group did not receive any treatment. All research experiment protocols adhered to the Principles of Laboratory Animal Care, and were approved by the Institutional Animal Care and Use Committee of Jilin University.

### Evaluation of kidney function

In our study, all animals were sacrificed on day 15. To determine the biochemical variables (e.g., urea nitrogen) using a Biochemistry Autoanalyzer, blood and urine were collected on days 8 (before treatment) and 15, respectively. Urinary levels of NAG and LZM were normalized by urinary creatinine, since it corrects for the filtered contribution of a given marker. To evaluate the effect of BMSCs and muscone on the accrementition and enlargement of tissue damage induced by gentamicin in the kidney, the kidney weight coefficient of each group was determined using the following algorithm: kidney weight coefficient  =  bilateral kidney weight (g) / body weight (g). Our previous study had showed that the kidney weight coefficient of normal rat was below 0.01 while average coefficient was over 0.02 in model group [34]. Therefore, this coefficient could be used for the evaluation of AKI model induced by gentamicin. For histological analysis, kidney tissue samples of each group were fixed with formalin, embedded in paraffin, sectioned to 5 µm-thick, and processed by hematoxylin and eosin (H&E) staining. Then the slides were reviewed blindly and scored with a semiquantitative scale evaluating changes found in AKI as the reference [55]. For each kidney, 100 cortical tubules from at least 10 different areas were scored, and avoid repeated scoring of different convolutions of the same tubule. The average histological score was used to valuate kidney tissue morphology. Higher scores represented more severe damage. On the other side, the samples fixed in glutaraldehyde were dehydrated in graded ethyl alcohols, and further embedded with Epon812. An ultrathin section was cut with an Ultracut E ultramicrotome, stained with uranyl acetate and lead citrate, and observed under transmission electron microscopy (TEM) as previously described [56], [57].

### Analysis of cell apoptosis

Apoptosis in the kidney tissue was evaluated by enzymatic labeling of DNA strand breaks using terminal deoxynucleotidyl transferase-mediated deoxyuridine triphosphate nick end-labeling (TUNEL) assay kit (KeyGEN) according to the manufacturer's instructions. The expression levels of apoptotic genes (Caspase 3, Fas, and Bax) and anti-apoptotic gene (Bcl-2) in the kidney tissues of each group were further detected using real-time qPCR and immunohistochemistry. The activated Caspase3 in kidney tissues was further detected with Caspase-3 colorimetric assay kit (KeyGEN) according to its manufacturer's instructions

### Real-time qPCR

For real-time qPCR analysis, kidney tissues (40 mg) or 3×10^6^ cells were homogenized in 1 mL of Trizol reagent (Invitrogen), and total RNA was extracted. RNA (2 µg) was reverse-transcribed using an RT–PCR kit (Takara, Japan), and qPCR was performed using a Thermal Cycler Dice™ Real-Time System and SYBR Green Premix EX Taq™ (Takara). GAPDH was used for qPCR normalization and all items were measured in triplicate. Primer sequences were obtained from the references [11], [34], [58]–[64].

VEGF forward (F) 5'-ACAGCTTTTTGCCTTCGAGCTA-3'

reverse (R) 5'-CATCAAAGCCCTTGTCGGGATA-3'

HGF forward (F) 5'-GGCTTTACTGCTGTACCTCC-3'

reverse (R) 5'-CAAATGCTTTCTCCGCTCT-3'

BMP-7 forward (F) 5'-AGACGCCAAAGAACCAAGAG-3'

reverse (R) 5'-GCTGTCGTCGAAGTAGAGGA-3'

IL-10 forward (F) 5'-GCAGGTGTCCCAAAGAAG-3'

reverse (R) 5'-TCAAAGGTGCTGAAGTCC-3'

TGF-β forward (F) 5'-CTGCTGACCCCCACTGATAC-3'

reverse (R) 5'-GTGAGCACTGAAGCGAAAGC-3'

TIMP-1 forward (F) 5'-ATTTGCACATCACTGCCTGC-3'

reverse (R) 5'-GGGATGGCTGAACAGGGAAA-3'

ET-1 forward (F) 5'-TCAGAGCAACCAGACACCGT-3'

reverse (R) 5'-CTTGGAAAGCCACAAACAGC-3'

Caspase 3 forward (F) 5'-GACAGTGGCATCGAGACAGA-3'

reverse (R) 5'-GAAAAGTGGCATCAAGGGAA-3'

Fas forward (F) 5'-CTCTGGAAGTGCATGCTGTAAGA-3'

reverse (R) 5'-GGTAGATGTCATTTGCGAAAGGT-3'

Bax forward (F) 5'-CTGCCAACCCACCCTGGT-3'

reverse (R) 5'-TGGCAGCTGACATGTTTTCTG-3'

Bcl-2 forward (F) 5'-TCTGTGGATGACTGAGTACCTGAAC-3'

reverse (R) 5'-AGAGACAGCCAGGAGAAATCAAAC-3'

MCP-1 forward (F) 5'-CAGAAACCAGCCAACTCTCA-3'

reverse (R) 5'-GTGGGGCATTAACTGCATCT-3'

RANTES forward (F) 5'-GGGCAGATGATTCTGAGACAAC-3'

reverse (R) 5'-CCAGGAATGAGTGGGAGTAGG-3'

MIP-2 forward (F) 5'-TTGTCTCAACCCTGAAGCCC-3'

reverse (R) 5'-TGCCCGTTGAGGTACAGGAG-3'

CXCR4 forward (F) 5'-TAGTGGGCAATGGGTTGGTAATC-3'

reverse (R) 5'-CTGCTGTAAAGGTTGACGGTGTA-3'

CXCR7 forward (F) 5'-TCACCTACTTCACCAGCACC-3'

reverse (R) 5'-ACATGGCTCTGGCGAGCAGG-3'

GAPDH forward (F) 5'-GCCAGCCTCGTCTCATAGACA-3'

reverse (R) 5'-AGAGAAGGCAGCCCTGGTAAC-3'

### Western blot

In our experiment, cells were harvested at the indicated times with RIPA lysis buffer (50 mM Tris–HCl, pH 7.4; 1% TritonX-100; 150 mM NaCl; 1% sodium deoxycholate; 0.1% SDS), including the phosphatase inhibitors (sodium orthovanadate, 2 mM) and protease inhibitor (0.5 µg/mL leupeptin, 0.1 µg/mL aprotinin, 0.6 µg/mL pepstatin A), for 30 min. After centrifugation at 12,000 rpm for 15 min, the protein content of cell lysates was determined using a BCA protein estimation kit (Pierce, USA), and bovine serum albumin was used as the standard. Equal amounts (15 µg) of protein were loaded per lane and electrophoresed in a 12% acrylamide gel, which was run at 120 V for 1 h. Protein transfer was performed using nitrocellulose for 1 h at 100 V. The primary antibodies used were anti-CXCR7 (1∶1000; Santa) and anti-CXCR4 (1∶1000; Abcam). Anti-rabbit or goat HRP and an Amersham ECL kit (GE Healthcare, USA) were used to detect protein. The band densities were quantified by densitometry (Quantity One v4.62).

### Immunohistochemistry

The cells or kidney tissues were fixed in 4% paraformaldehyde in PBS. The kidney tissues of each group were embedded in paraffin and sliced into 5 µm-thick sections. For immunohistochemistry, the primary antibodies used were anti-CK-18 (1∶50, Bioss), anti-Caspase 3 (1∶100, Abcam), anti-Fas (1∶50, Santa), anti-Bax (1∶50, Santa), anti-Bcl-2 (1∶100, Abcam), and anti-BrDU (1∶100, Abcam) polyclonal antibodies. After 12 h of incubation at 4°C, the samples were washed three times with PBS and processed using an SABC kit and DAB solution. Finally, the sections were observed using Axio Scope A1 (Zeiss, Germany) with AxioCAM MRc5 (Zeiss), and processed with AxioVision software (Zeiss). The color intensity was measured using Motic Image Advanced 3.2.

### Evaluation of inflammatory components

After the therapy, the inflammatory components in the kidney tissues of each group were detected with qPCR and ELISA. For the ELISA assay, kidney tissues (200 mg) were homogenized with PBS solution and the homogenate was centrifuged at 3000 rpm for 20 min. The supernatant was collected for ELISA. Herein, monocyte/macrophage chemotactic protein-1 (MCP-1), IL-10, regulated upon activation normal T cell expressed and presumably secreted (RANTES) and Macrophage inflammatory protein 2 (MIP-2) were examined for evaluation of inflammatory reaction.

### Cell migration assays

Both the scrape-healing assay and transfilter assay were performed as described in the literature [33]. In the scrape-healing assay, BMSCs were seeded at 8×10^4^ cells/well in 24-well plates and allowed to reach confluence. The cells were then switched to DMEM/F12 medium containing 2% FBS and incubated with muscone (0.3, 1.0, and 3.0 mg/L) for 24 h. The monolayers were then scratched by the tip of a 1ml pipette, washed with PBS, then observed under the microscope to determine the wound distance used for counting, and further incubated in the medium containing 2% FBS for 24 h. Finally, the images of cell samples were obtained for further analysis, and four random fields were counted for each group. For transfilter assay, BMSCs were treated as scrape-healing assay and seeded on the upper side of a porous polycarbonate membrane (pore size: 8 µm; Euroclone SPA, Italy) in co-culture with RTECs. RTECs were seeded at 9×10^4^ cells/well in a 24-well plate. After 24 h, cells were incubated with DMEM/F12 plus 2% FBS alone or in the presence of 5 µM cisplatin for 6 h. After cisplatin removal, the RTECs were washed and co-cultured with BMSCs. After 36 h, the cells at the upper side of the filter (unmigrated cells) were mechanically removed. Cells that had migrated to the lower side of the filter were fixed for 30 min in 4% paraformaldehyde and further stained with crystal violet. The number of cells in six random fields was counted for each filter. We also tried to induce the RTECs damage with gentamicin (10 U/ml, 30 U/ml and 100 U/ml). However, the damaged RTECs could not induce the cell migration effectively. Therefore, cisplatin was used in our study as the reference [33]. To investigate the influence of cell proliferation on the migration, mitomycin C was used to block proliferation. In our study, BMSCs incubated with 10 mg/mL mitomycin C (Sigma) for 60 min, were used for transfilter assay as the reference [65]. In addition, for further exploring the regulation of muscone on cell migration, BMSCs exposed to CXCR4 and CXCR7 neutralizing antibody (10 µg/ml), were used for scrape-healing assay, as well as transfilter assay.

### Statistical analyses

The results are expressed as means ± SEM, and statistical analysis was performed using SPSS 17.0. The differences among groups were analyzed by one-way ANOVA followed by t-test. *P*<0.05 was considered statistically significant.

## Supporting Information

Figure S1 **Effect of muscone on RTEC bioactivity** ***in vitro*** **.** A: Effect of muscone on RTEC proliferation. Proliferation index (the absorbance of experimental group − the absorbance of blank group) was measured using CCK-8. B: Effect of muscone on RTEC secretion. To evaluate the secretory function, the secretion or cytokine expression level of normal BMSCs without muscone treatment was 1.0 for each cytokine. Cytokine expression in RTECs detected using qPCR is shown on the left, and the RTEC secretory function evaluated using ELISA is shown on the right. Similar results were obtained in at least three independent experiments. Results are expressed as mean ± SEM. A *t*-test was used to compare the various groups, and *P*<0.05 was considered statistically significant. *P<0.05 compared with the normal RTEC group without any muscone treatment.
(PDF)

Figure S2 **Expression of apoptotic genes and anti-apoptotic gene in the kidney tissues of each group.** The expression levels of apoptotic genes (Caspase 3, Fas, and Bax) and anti-apoptotic gene (Bcl-2) in the kidney tissue were also detected using immunohistochemistry. A representative in the kidney tubules, kidney glomeruli, and collecting tubules close to the average level of each group is shown for each group. Scale bar corresponds to 100 µm.
(PDF)

Figure S3 A: Transmigration of muscone-treated BMSCs toward the cisplatin-injured RTECs. BMSCs co-cultured with healthy RTECs or without RTECs were used as the control group. *P<0.05 compared with the normal BMSC group without any muscone treatment. B: Effect of incubation time on cell migration in transfilter assay. *P<0.05 compared with the normal BMSC group without any muscone treatment after same incubation time. C: Effect of cell proliferation on cell migration in transfilter assay. BMSCs treated with mitomycin C were used for transfilter assay, and no significant difference existed between the normal BMSCs and the mitomycin C treated BMSCs.
(PDF)

Figure S4 **Preparation and treatment of rat AKI model.** The rat AKI model was induced with gentamicin and further treated with dexamethasone, muscone, and stem cells.
(PDF)

Table S1 **The absolute values in proliferation indexes of each group.** BMSCs were treated with muscone at different concentrations (0.3, 1.0, and 3.0 mg/L) Proliferation index (the absorbance of experimental group − the absorbance of blank group) on day 0, day 1, day 2 and day 3 was measured using CCK-8.
(PDF)

Table S2 **Urinary creatinine during the therapy process.** I: urinary creatinine for characteristics of AKI model; II: urinary creatinine after the therapy.
(PDF)
