# Supplementary figures and images for: Administration of BMSCs with Muscone in Rats with Gentamicin-Induced AKI Improves Their Therapeutic Efficacy
Source: PLoS One. 2014 May 13;9(5):e97123. doi: 10.1371/journal.pone.0097123 (PMC4019657; doi:10.1371/journal.pone.0097123)

Figure S1

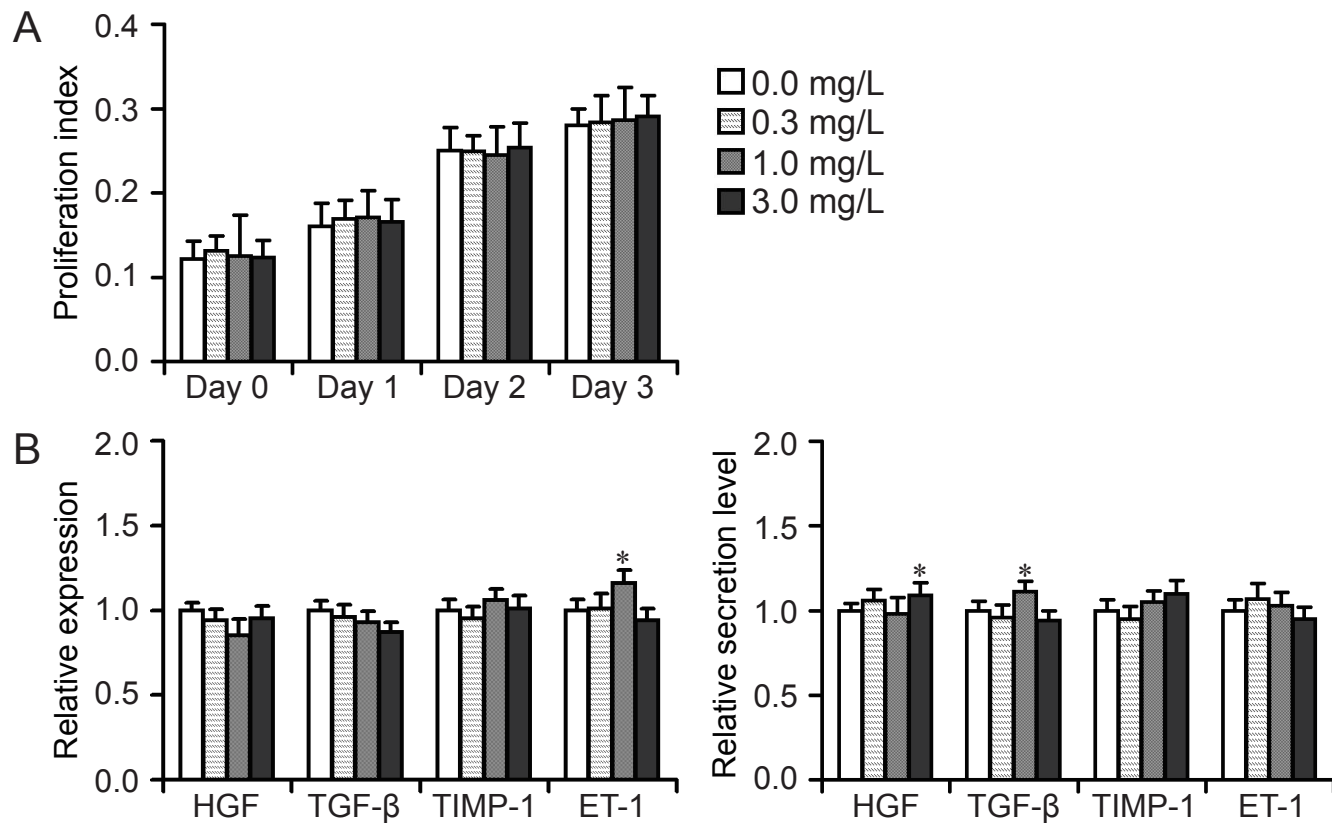

Supplement: Figure S1 — Effect of muscone on RTEC bioactivity in vitro . A: Effect of muscone on RTEC proliferation. Proliferation index (the absorbance of experimental group − the absorbance of blank group) was measured using CCK-8. B: Effect of muscone on RTEC secretion. To evaluate the secretory function, the secretion or cytokine expression level of normal BMSCs without muscone treatment was 1.0 for each cytokine. Cytokine expression in RTECs detected using qPCR is shown on the left, and the RTEC secretory function evaluated using ELISA is shown on the right. Similar results were obtained in at least three independent experiments. Results are expressed as mean ± SEM. A t-test was used to compare the various groups, and P<0.05 was considered statistically significant. *P<0.05 compared with the normal RTEC group without any muscone treatment. (PDF) [file pone.0097123.s001.pdf]

Figure S2

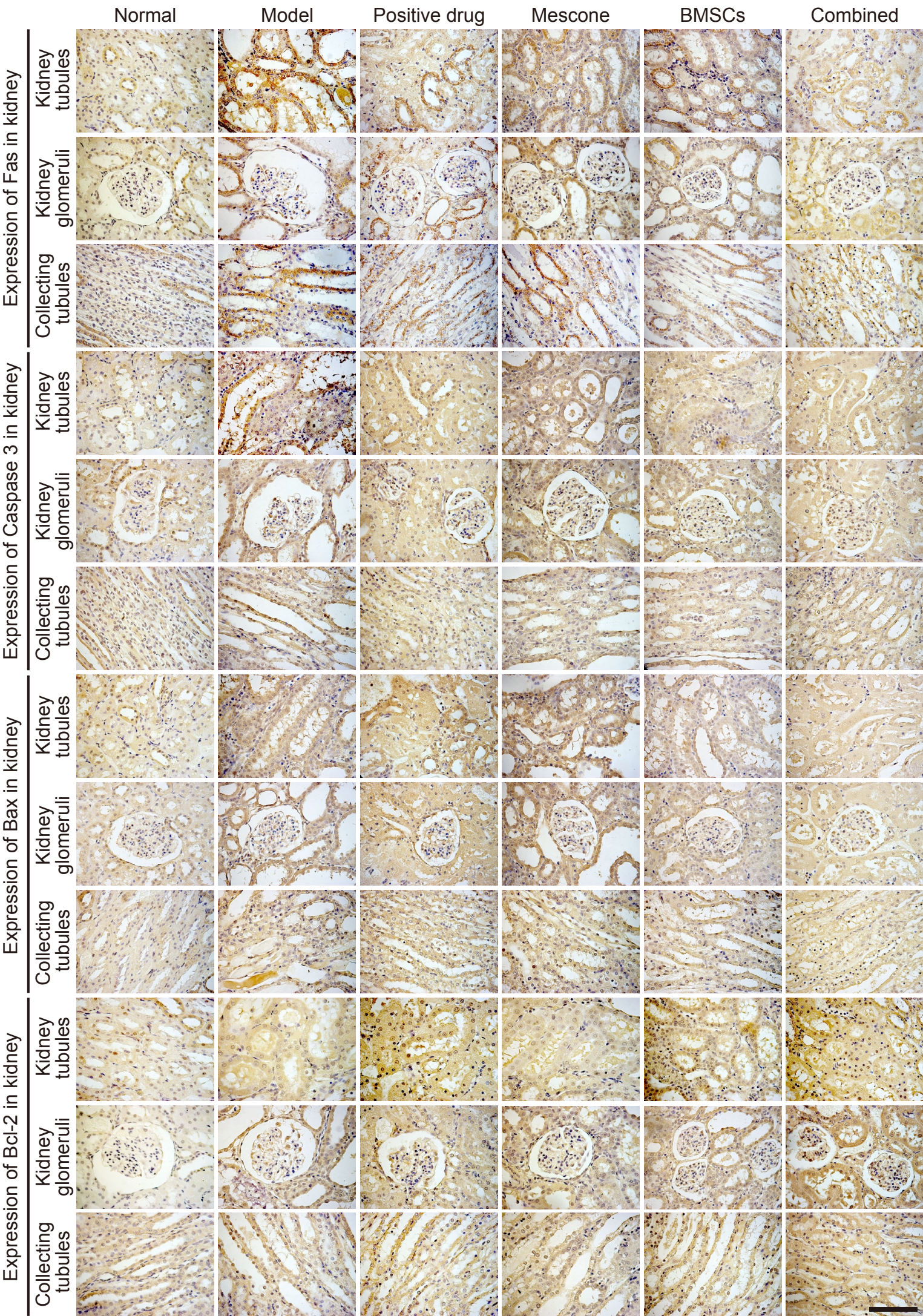

Supplement: Figure S2 — Expression of apoptotic genes and anti-apoptotic gene in the kidney tissues of each group. The expression levels of apoptotic genes (Caspase 3, Fas, and Bax) and anti-apoptotic gene (Bcl-2) in the kidney tissue were also detected using immunohistochemistry. A representative in the kidney tubules, kidney glomeruli, and collecting tubules close to the average level of each group is shown for each group. Scale bar corresponds to 100 µm. (PDF) [file pone.0097123.s002.pdf]

# Figure S3

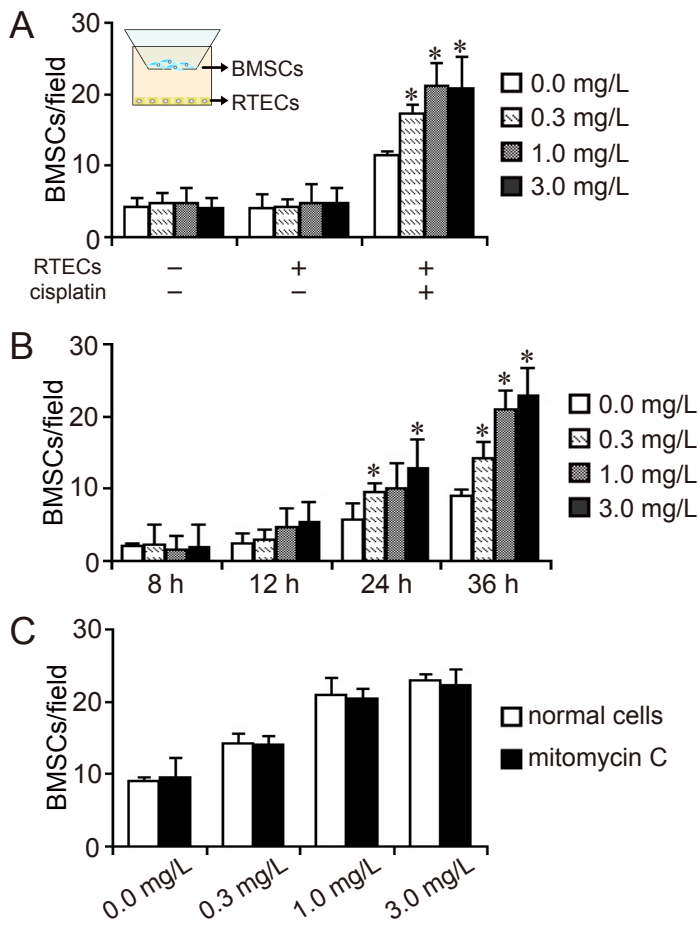

Supplement: Figure S3 — A: Transmigration of muscone-treated BMSCs toward the cisplatin-injured RTECs. BMSCs co-cultured with healthy RTECs or without RTECs were used as the control group. *P<0.05 compared with the normal BMSC group without any muscone treatment. B: Effect of incubation time on cell migration in transfilter assay. *P<0.05 compared with the normal BMSC group without any muscone treatment after same incubation time. C: Effect of cell proliferation on cell migration in transfilter assay. BMSCs treated with mitomycin C were used for transfilter assay, and no significant difference existed between the normal BMSCs and the mitomycin C treated BMSCs. (PDF) [file pone.0097123.s003.pdf]

# Figure S4

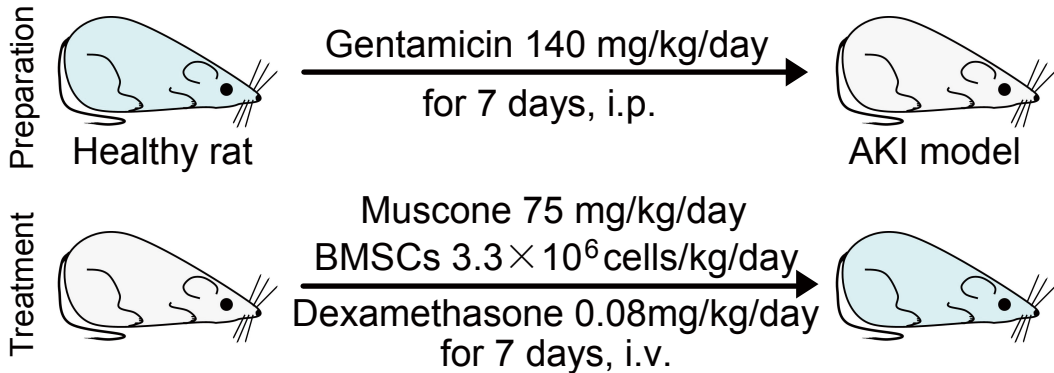

Supplement: Figure S4 — Preparation and treatment of rat AKI model. The rat AKI model was induced with gentamicin and further treated with dexamethasone, muscone, and stem cells. (PDF) [file pone.0097123.s004.pdf]
