# Supplementary material for: Administration of BMSCs with Muscone in Rats with Gentamicin-Induced AKI Improves Their Therapeutic Efficacy
Source: PLoS One. 2014 May 13;9(5):e97123. doi: 10.1371/journal.pone.0097123 (PMC4019657; doi:10.1371/journal.pone.0097123)
Supplement: Table S1 — The absolute values in proliferation indexes of each group. BMSCs were treated with muscone at different concentrations (0.3, 1.0, and 3.0 mg/L) Proliferation index (the absorbance of experimental group − the absorbance of blank group) on day 0, day 1, day 2 and day 3 was measured using CCK-8. (PDF) [file pone.0097123.s005.pdf]

Table S1. The absolute values in proliferation indexes of each group

Time	0.0 mg/L	0.3 mg/L	1.0 mg/L	3.0 mg/L
Day 0	0.202±0.041	0.210±0.042	0.223±0.031	0.214±0.043
Day 1	0.321±0.053	0.378±0.050	0.441±0.040	0.433±0.051
Day 2	0.521±0.051	0.602±0.043	0.656±0.043	0.667±0.055
Day 3	0.600±0.034	0.667±0.051	0.703±0.035	0.711±0.043
