# Supplementary material for: Administration of BMSCs with Muscone in Rats with Gentamicin-Induced AKI Improves Their Therapeutic Efficacy
Source: PLoS One. 2014 May 13;9(5):e97123. doi: 10.1371/journal.pone.0097123 (PMC4019657; doi:10.1371/journal.pone.0097123)
Supplement: Table S2 — Urinary creatinine during the therapy process. I: urinary creatinine for characteristics of AKI model; II: urinary creatinine after the therapy. (PDF) [file pone.0097123.s006.pdf]

Table 2. Urinary creatinine during the therapy process

Group	Urinary Creatinine (mM)	
	I	II
normal group	3.6±0.5	3.5±0.7
model group	2.1±0.7	2.3±0.4
positive drug group		3.0±0.6
muscone group		2.8±0.7
BMSCs group		3.1±0.6
combined group		3.2±0.4
